# The experienced route to cognitive health: Cognitive recovery in persons with prior stress-related Exhaustion disorder

**DOI:** 10.1186/s12888-025-06713-7

**Published:** 2025-04-14

**Authors:** Andreas Nelson, Ingela Aronsson, Maria Tillfors, Anna Stigsdotter Neely, Hanna M Gavelin

**Affiliations:** 1 Department of Social and Psychological Studies, Karlstad University, Karlstad, Sweden; 2 Department of Psychology, Umeå University, Umeå, Sweden; 3 Department of Social Sciences, Technology and Arts, Department of Health, Education and Technology, Luleå University of Technology, Luleå, Sweden; 4 Department of Public Health and Clinical Medicine, Section for Sustainable Health, Umeå University, Umeå, Sweden

**Keywords:** Cognition, Cognitive recovery, Stress-related exhaustion, Exhaustion disorder, Clinical burnout

## Abstract

**Background:**

People diagnosed with stress-related exhaustion disorder report high levels of cognitive symptoms. This study aimed to explore how persons diagnosed with Exhaustion disorder (ED) experienced cognitive functioning and recovery 6-10 years after participating in a rehabilitation programme. Specifically, it investigated the experiences of current functioning, change over time, and what had been barriers or facilitators for cognitive recovery.

**Methods:**

Semi‐structured interviews were conducted for 38 persons previously diagnosed with ED (Mean age: 52; Females: 32) and explored using thematic analysis.

**Results:**

The analysis resulted in four themes: “’It’s different now’: Remaining cognitive symptoms”, “The bigger picture: Cognitive recovery in context”, “Overcoming challenges: Strategies for coping with cognitive symptoms”, and “The approach towards cognition matters”. The participants’ experiences varied but included descriptions on how cognitive functioning had become better with some remaining symptoms. These difficulties were reported across cognitive domains, yet often centred around upholding executive control. Cognitive recovery was seen in the context of overall well-being and recovery which differed between the participants. Facilitators and barriers thus varied between persons, and included both restorative and compensatory strategies, external conditions, the degree of worry, and development of a more acceptant or self-compassionate view on cognition and oneself.

**Conclusions:**

The results show that cognitive recovery in ED is multifaceted. 6-10 years after rehabilitation, experiences included improvement of everyday cognitive functioning, but also lingering challenges, often related to maintenance of executive control. Recovery was influenced by factors such as general well-being, restorative or compensatory strategies, worrying, or the adaptation of more accepting or self-compassionate perspectives. The findings indicate a need for tailored, person-centred approaches to supporting cognitive recovery.

**Trial registration:**

Participants were recruited as part of the Rehabilitation for Improved Cognition (RECO) trial (ClinicalTrials.gov: NCT03073772, date of registration: 8 March, 2017). This study was preregistered on the Open Science Framework (osf.io: https://doi.org/10.17605/OSF.IO/S2W6X).

**Supplementary Information:**

The online version contains supplementary material available at 10.1186/s12888-025-06713-7.

## Introduction

Stress-related illness is a growing concern in western societies. In Sweden, Exhaustion disorder (ED), i.e. a diagnosis describing a sequela to long-term stress exposure often referred to as clinical burnout, is now one of the most common reason behind sick leave [23, 24]. This construct was introduced in 2005 to the Swedish version of the International Classification of Diseases (ICD-10-SE; [55]) with the core symptom being physical and mental exhaustion as a response to identifiable stressors. As ED is a relatively novel construct, there is a lack of scientific consensus about the overall conceptualisation of the disorder as well as of treatment recommendations and symptomology, including cognitive dysfunction [43]. The current diagnostic criteria (Appendix 1, Additional file 1) comprise memory or concentration difficulties and, accordingly, a growing body of research has found patients with ED to report high levels of cognitive problems in everyday life [47], in the research literature often referred to as subjective cognitive complaints (SCC:s; e.g., [35]). Moreover, cognitive testing has provided evidence that compared to healthy controls, patients with ED perform worse in tasks tapping episodic memory, working memory, executive functions, attention, processing speed and fluency, broadly suggesting cognitive control deficiency [26], possibly remaining over time [37]. However, differences in test performance between ED patients and controls are often small to moderate, and studies have presented no or low correlations between test performance and SCCs (e.g., [36, 47, 66]). Notably, weak or non-existent associations between subjective measures and test-results have been highlighted in several other clinical (e.g., [7, 50, 54, 60]) and non-clinical (e.g., [10, 47]) groups and are hence not unique to ED. This suggests that cognitive tests and self-reported everyday difficulties may not be interchangeable and markers of different aspects of cognition [59], indicating a need to further understand the subjective experience of cognitive problems commonly reported by persons diagnosed with ED.

Previous studies have studied SCCs quantitively, using either standardized questionnaires (e.g., [21, 22, 36, 47, 66]) or single items [20, 36] targeting everyday cognitive slips and failures. This work has shown that the overall level of SCCs (i.e., total scores on questionnaires) is higher in the ED population than in healthy control groups, and that self-report indicates relatively more cognitive difficulties compared to cognitive test results [47]. Yet, comparatively few studies have investigated the experience and type of cognitive dysfunction in ED from the first-hand perspective. Qualitative reports focussing broadly on ED symptomology have revealed patients to experience cognitive dysfunction as the most “unbearable” of the symptoms associated with the condition [42] and that cognitive problems may remain a decade after the onset of the disorder, including difficulties with memory, concentration, and hyper-sensitivity towards external stimuli [21]. Using the Prospective and Retrospective Memory Questionnaire (PRMQ; [13]) our group has previously reported that people with ED report relatively more memory failures in “self-cued” memory tasks (i.e., under circumstances where memory is not aided by reminders in the environment), perhaps reflecting the executive difficulties suggested by cognitive testing [47]. Yet, more information is needed on how persons who have suffered from ED would describe these or other cognitive symptoms themselves, including how the problems may have changed over time and what has been helpful or hindering in the recovery process. With a specific focus on cognition, this study interviewed persons previously diagnosed with ED who have undergone multimodal, cognitive behavioural therapy (CBT)-based, rehabilitation. The aims were to explore how they experienced cognitive functioning and recovery at the time of the interview, 6-10 years after participating in the rehabilitation programme, with the following research questions:

1. What were the experiences of current cognitive functioning, at the time of the interview?
2. What were the experiences of change in cognitive functioning over time, during the recovery process?
3. What were experienced as barriers or facilitators of cognitive recovery?

## Methods

### Design and theoretical considerations

Individual semi-structured interviews were conducted in order to better understand the experience of persons diagnosed with ED. The epistemological standpoint of this study adheres to critical realism, acknowledging the impact of society and the researchers’ subjective perspective on the research process while still assuming the existence and importance of external reality [41]. The interviews were interpreted using thematic analysis in the specific form of Template analysis [6], as further described below. This theoretically flexible method allows a reflexive, largely inductive stance in terms of coding while still enabling the inclusion of themes stated *a priori*, in this case domains of cognitive abilities. In doing so, the technique fits a theoretical position in between a strictly positivist viewpoint and the more inductive and subjective stance of for instance the Reflexive Thematic Analysis approach advocated by Braun and Clarke [5]. Reporting follows the guidelines from the Consolidated Criteria for Reporting Qualitative Research, COREQ [58].

### Participants

This study was a part of the larger Rehabilitation for Improved Cognition (RECO) project, a longitudinal randomized controlled clinical trial examining the effects of computerized cognitive training or aerobic training on cognitive function, psychological health and work ability for people with ED [25, 27]. All participants had in addition to the training interventions participated in a 24-week multimodal stress rehabilitation programme, comprising elements of CBT, physiotherapy, vocational rehabilitation and regular appointments with a physician. The recruitment was conducted between April 2010 and June 2013. Inclusion criteria were: (1) ED diagnosis, confirmed by a physician and a psychologist; (2) 18–60 years old; (3) currently employed; (4) considered by a physician and a psychologist to be suitable for group-based stress rehabilitation; (5) no known abuse of alcohol or drugs; (6) not in need of more urgent treatment; and (7) not participating in other interventional study. Patients with relevant diagnoses in addition to ED (e.g., neurological or chronic psychiatric diagnoses) that required special care and treatment adjustments were not considered suitable for the standardized group-based stress rehabilitation, and therefore not included in this study.

The CBT focussed largely on stress management, including strategies and activities for aiding sleep and recovery, and increased awareness on how emotions influence stress-related problems. Treatment effects were evaluated using cognitive tests and questionnaires at multiple timepoints: immediately after the interventions, after approximately 1 year and, again, 4.5 years after treatment. At the initial stage, the RECO-project included 132 participants of which 56 participated in the 4.5-year follow-up. In order to capture the broadest possible spectrum of views and information, all 56 participants were invited to participate in the 6-10 years interviews. Of these, 38 participants were included and completed the interviews. For the included 38 participants, the time that had passed ranged between 6 and 10 years, with an average of 8 years. They did not differ from neither the 18 persons from the 4.5-year follow up not included in this study, nor from the initial sample at the pre-intervention assessment, with respect to demographic (age, sex, education level, verbal ability) and psychological (self-rated symptoms of anxiety, depression and burnout) characteristics at baseline (data not shown). The flow of participants is visually presented in Appendix 2, Additional file 1.

In order to provide a quantitative description of the sample, questionnaires were filled out prior to the interviews. The Shirom-Melamed Burnout Questionnaire (SMBQ; [45]) assessed symptoms of burnout, specifically with respect to fatigue, tension, listlessness, and cognitive weariness. Answers were rated on a seven-point scale and the results summarized as the mean score of all 22 items, with a score over 4.4 indicating severe burnout. The Exhaustion Disorder scale (s-ED; [31]) was used to assess the rate of ED-symptoms and generates an indication of whether ED-criteria are fulfilled. The Hospital Anxiety and Depression Scale (HADS; [65]) measured symptoms of depression and anxiety on separate scales ranging from 0-21; a score over 10 was interpreted as probable occurrence of clinical depression or anxiety. A single question (“Assume that your work ability at its best has a value of 10 points. How many points would you give your current work ability?”) was included from the Work Ability Index (WAI; [16] in order to assess self-rated work ability; the answers ranged from 0 = “cannot work at all” to 10 = “work ability at its best”. Moreover, one question about self-rated general health (“In general, how would you say your health is: poor, fair, good, very good, excellent?”) was included from the SF-36 [52], as was one question on perceived recovery from ED (“How recovered do you feel after your previous period of exhaustion?”), ranging from 0 (“not recovered”) to 7 (“fully recovered”). Table 1 shows the sample characteristics. Notably, a majority of participants reported that they were in good or excellent health. However, about a third of the participants perceived that they were not fully recovered from exhaustion and still fulfilled the ED criteria or were above the cutoff of severe burnout, according to the s-ED and SMBQ, respectively. A majority of the participants, 32 out of 38, were women. The mean age was 52, ranging from 34-67.

**Table 1:** Participant characteristics at time of the interview

**Variable**	**Mean (SD) or N (%)**
**Age**^**a**^	
Mean (SD)	51.9 (7.9)
Range	34-67
**Gender**^**a**^	
f/m	32/6 (84%/16%)
**SMBQ**^**b**^	
Mean (SD)	3.7 (1.2)
Above cut-off (>4.4)	10 (27%)
**s-ED**^**b**^	
Meet the criteria	13 (35.1%)
**HADS-A**^**c**^	
Mean (SD)	7.5 (3.9)
Above cut-off (>10)	7 (19.4%)
**HADS-D**^**c**^	
Mean (SD)	4.0 (3.1)
Above cut-off (>10)	1 (2.7%)
**Perceived work ability**^**c**^	
Mean (SD)	7.1 (1.6)
**Perceived general health**^**b**^	
Excellent/Very good	8 (21.6%)
Good	22 (59.5%)
Fair/Poor	7 (18.9%)
**Perceived degree of recovery from ED**^**b**^	
1-2 (not recovered)	0 (0%)
3-5	30 (81.1%)
6-7 (fully recovered)	7 (18.9%)
**Percentage working hours**^**d**^	
0-24%	0 (0%)
25-49%	1 (2.6%)
50-74%	2 (5.3%)
75-99%	5 (13.2%)
100-%	22 (57.9%)
Retirement	2 (5.3%)
Missing	6 (15.8%)

*Note*. *SMBQ* Shirom-Melamed Burnout Questionnaire, *s-ED* Self-rated Exhaustion Disorder, *HADS* Hospital Anxiety and Depression Scale. Number of respondents varies for different variables due to missing data. ^a^*n*=38, ^b^*n*= 37, ^c^*n*=36, ^d^based on information reported in the memory aid

### Procedure

The interviews were conducted during 2019 and 2020 by a member of the research team (IA). She had previously met some of the participants during earlier phases of the RECO-project, conducting neuropsychological tests and/or providing guidance on how to do the cognitive training. IA is also experienced as a clinical psychologist at the Stress Rehabilitation Clinic in Umeå but had not participated in any psychotherapeutic treatment of the participants of this study. The interviews were held at the stress rehabilitation clinic (*N*=31) or otherwise, in cases where participants lived far away from the clinic, over video link (*n*=7). In order to assist recollection and reflection, a memory aid was handed out prior to the interviews with the instructions to specify key life events that had hindered and helped recovery after treatment, based on the categories: “Work”, “Family”, “Social network”, “Leisure”, “Healthcare”, “Other”, “Things I have done myself” (Appendix 3, Additional file 1). The interviews were audio recorded and transcribed verbatim by secretaries hired by the project. The analyses are based on the transcribed interviews, exclusively. A semi-structured interview guide was used to aid the interviews which lasted between 52 and 72 minutes. The principle focus of the current study was a section of questions specifically targeting cognitive functioning. In summary, these questions were: “Based on the survey (then follow up): How is your memory/ concentration/ mental fatigue functioning today?”; “If improvement, what has been helpful?”; “Any obstacles/things that made it difficult?”. Notably, cognitive recovery was not the sole focus of the interviews as the guide also included more general questions concerning recovery behaviours and recuperation from ED (see Additional file 1, Appendix 4 for the full set of questions and potential follow-up queries). Analysis of the remaining questions have recently been presented in a separate paper, focussing more broadly on the experience of the recovery process in people previously diagnosed with ED [2]. In cases where the participants answered these remaining questions and explicitly addressed relevant topics with respect to the aims of the current study, the answers were coded and included in the results.

### Data analysis

#### Method and theoretical considerations

Interview data was analysed using thematic analysis in the specific form of Template analysis, following Brooks et al [6]. This method is concentrated around the development of a hierarchically structured template of coded themes and was chosen as it is theoretically flexible, allowing inclusion of themes stated a priori, aiding development of an initial, preliminary version of the template [6, 44]. The rational for this decision was that since most authors of this study (HM, AN, ASN, IA) have backgrounds in clinical neuropsychology (MT’s research background is primarily in clinical psychology), we argue that it is important to acknowledge the influence of pre-existing views of cognition on our interpretation of the data. Hence, our (the authors’) views and definitions of some common cognitive functions and domains (episodic memory, executive functions, attention and concentration, and mental speed, as well as some subdomains) were discussed before, and continuously during, the early phases of analysis. They were summarized in a document together with some examples to aid the initial coding process (Additional file 1, Appendix 5). No single theoretical framework was used to create this document, which instead drew from several sources (e.g., [13, 18, 30]). Importantly, the cognitive areas specified before coding and added to the template were regarded as tentative and continuously altered during the course of data analysis. It is also worth noting that it only comprised a priori statements regarding type of cognitive functioning and only a selection of the cognitive areas that could have been derived from theory. In other words, the chosen cognitive concepts stated a priori were a starting point, covering some of the cognitive areas common in the research literature. It was not intended to cover all possible topics or themes that were likely to appear in the data or from the research questions. Since we have previously presented results showing that ED patients reported relatively more “self-cued” than “environmentally cued” memory failures when filling out the PRMQ [47] we were specifically interested to see if such pattern would emerge when analysing the interview data in the current study. We therefore added the memory concepts described in the PRMQ (prospective, retrospective, self-cued, environmentally cued, short-term and long-term memory) to the a priori template, as subdomains of episodic memory. Notably, not all of these domains were included in the final template or thematization as they did not readily correspond with the participants’ reports.

#### Analysis

Following the creation of the a priori template, the analysis was completed through the six steps proposed by Brooks et al. [6]: (1) becoming familiar with the accounts to be analysed, (2) carrying out preliminary coding of the data, (3) organizing the emerging themes into meaningful clusters, and beginning to define how they relate to each other within and between these groupings, (4) defining an initial coding template, (5) applying the initial template to further data and modifying as necessary, (6) finalizing the template and applying it to the full data set. In practice, the template was altered successively through several iterations where the full data set was primarily read, analysed and structured, by the first author who discussed and received recurrent feedback from the other authors during this process. Data analysis was finalized when all authors had agreed on the thematization and structure of the template, as well as on the interpretation and conclusions of the results. The a priori and the final template are presented in the supplementary material (Additional file 1, Appendices 6 and 7). The coding of the interviews was performed using the NVivo software, versions 12 and 14. Notably, stating the frequency of themes or content was not a main aim of the thematic analysis. However, in the results, the wording used for summarizing the number of participants having expressed a certain theme was defined prior to analysis (“A few”: 0-5 participants, “Some”: 6-10, “Several”: 11-20, “Many”: 21-30, “Most”: 30-34, “Almost all”: 35-38). The names and identification numbers of the participants used in the RECO-trial were replaced with a code in form of a letter or letter-number combination (e.g., Participant A or Participant C2). The presented quotes were translated from Swedish to English and slightly altered in order to enhance clarity and readability, for instance by removing punctuation, altering idiomatic expressions and non-relevant words. In some cases, the quotes’ content such as setting and personal examples were modified in order to make identification of the participants more difficult.

## Results

The analysis resulted in four top-level themes, each comprising subthemes. The first theme, “’It’s different now’: Remaining cognitive symptoms”, summarizes how 6-10 years after treatment, cognitive functioning was different. Although functioning was improved, remaining problems varied in level and ranged over multiple cognitive domains, but were still centred around difficulties with upholding concentration and executive control over time. The second theme, “The bigger picture: Cognitive recovery in context”, highlights how cognitive functioning was seen in tandem with stress and overall health, and that lifestyle changes facilitating recovery from ED also affected cognitive recovery. The third theme, “Overcoming challenges: Strategies for coping with cognitive symptoms”, displays how cognitive functioning was aided by the use of various strategies, including ways of compensating for difficulties or by challenging them; or by optimizing the conditions under which cognitive tasks were performed. The fourth theme, “The approach towards cognition matters”, describes how some participants still found cognitive problems frightening or distressing and how others had started to approach them with a more accepting, self-compassionate attitude which had helped facilitate cognitive functioning and recovery. Table 2 presents an overview of the themes, which are further explained and elaborated below.

**Table 2:** Overview of the coding template’s two highest levels of themes

**“It’s different now”: Remaining cognitive symptoms**	**The bigger picture: Cognitive recovery in context**	**Overcoming challenges: Strategies for coping with cognitive symptoms**	**The approach towards cognition matters**
Changes in cognitive functioning	Inner and outer barriers for cognitive functioning	Individual strategies	Distress relating to cognitive functioning
Type of remaining problems	Cognitive functioning is facilitated by general recovery from ED	Optimizing outer conditions	Acceptance, self-compassion, and -prioritization: A shift in perspective facilitates cognitive functioning

*Note.* Top-level themes are bolded

### Theme 1: “It’s different now”: Remaining cognitive symptoms

#### Changes in cognitive functioning

When reflecting on the present situation, most participants noted that compared to when at its worst, cognitive functioning was markedly improved*.* The degree of cognitive recovery varied between participants, with some describing their current functioning as plain better. More frequently, however, the participants reported lingering cognitive symptoms of varying severity. Several persons experienced that the cognitive difficulties were intermittent or that it did not have a substantial impact on everyday functioning, whereas others experienced them as more persistently challenging:

Participant R: It’s like nothing compares to when I was ill the first time, because it was so extreme.

Participant D2: I can do everything today, whatever I want […]. It’s amazing considering how bad it was during a year or so when I couldn’t manage anything.

Participant K: I can concentrate during longer periods now and process information in a better way. I’m not quite there with reading fiction yet, but at least I can listen to books.

Participant J: These difficulties are just there; I’ve had them for so many years and they’ve become normal to me [...]. But I know intellectually that it’s not ok or normal and that it wasn’t like this before. It’s like a handicap that I’ve somehow learnt to deal with as good as I can.

#### Type of remaining problems

The participants differed in the type of cognitive problems that were experienced at the time of the interview. Across persons, cognitive difficulties were expressed in multiple cognitive domains (sample quotes for each of the domains mentioned in this section are presented in Table 3). However, despite the range of specific cognitive problems reported, difficulties in exerting and upholding executive control was a common factor across domains. For instance, regarding episodic memory, failures were noted in both retrospective and prospective memory tasks. Yet, as further elaborated on below, many participants reported that memory functioning was dependent on the use of external memory aids, indicating difficulty with self-cued memory. Furthermore, participants emphasized difficulties with learning new information as opposed to accessing well-learnt knowledge and more automated abilities*,* signifying suboptimal self-initiated processing and executive functioning [18]. Executive problems were moreover reported with respect to inhibition of stimuli or switching between different task (i.e., with cognitive flexibility). Several participants also noted problems in maintaining attention and working memory performance, particularly over longer periods of time, often concurrent with a heightened sensitivity to sensory input.

**Table 3:** Examples of remaining cognitive symptoms per domain

Cognitive domain	Example quote
Episodic memory	
Prospective memory	“Someone says that he’ll be late Friday. That’s forgotten. The hard drive is full”. (Participant I)
Retrospective memory	“I may have done or put something somewhere and can’t remember it. Sometimes, I can rewind memory and remember. Other times, it’s like it never happened”. (Participant B)
Self-cued memory	“If I know that something is important to memorize, then I’ll have to think about it actively […] For example, in order to remember dentist appointments, I note them in the calendar. Sometimes, I miss it anyway, so I’ve placed a toothbrush on the bureau in the hallway. That is, I visualize to remember things. If I’ve been somewhere, I try to remember the environment around me: what kind of weather or what year it is, or the people that’s there. That kind of stuff can help me remember things in the future”. (Participant J)
New learning	“How can I get a job now that I can’t learn new things? It doesn’t stick. […] If someone explains how to do something, 2 seconds - or 2 minutes - later it’s gone again”. (Participant G2)
Attention/ concentration	
General concentration	“I can read three pages three times and still not remember that I’ve read it. I just see the text”. (Participant D)”
Vigilance	“At work, during long administrative meetings, you need to sit there and listen, and then to be in groups writing post-its, expressing your opinions and summarizing. You need to work and be present and focused the whole day, which I can’t cope with. During long meetings, I start to fiddle with my cell phone or go to the rest room or something like that. I’m affected in not having stamina. It’s like I can perform well but not for too long”. (Participant B2)
Executive functions	
Inhibition	“If I deliberately make up my mind and really focus and get rid of all external impressions – which is energy consuming – it [the ability to concentrate] kind of works. But I have to make an active choice. Normally, it doesn’t really stick, thought-wise, but sometimes, if I decide to allocate energy, concentration and put everything else aside, then it might work pretty well”. (Participant B).
Cognitive flexibility	“Let’s say I’m cooking. I need to be totally focussed on what I am doing. Then someone comes in and says something to me and I just feel 'God what was I doing? Did I do it wrong? Did I really put milk in whatever needed milk?'" (Participant Y).
Working memory	“I lose the thread easily. When saying something, I start asking myself: “Where the heck am I going with this?” So even as I’m thinking, I easily lose track of where I’m going. And if I’m interrupted, I can’t always remember what I was just saying”. (Participant Z).
Higher-order EF/ problem solving	“I don’t have the ability to think. I do work that involves analysis and I can’t do it, it’s like it just stops. I recently tried to increase my work hours which now resulted in me staying a week home from work. I got extremely dizzy and have difficulties understanding ordinary, simple things at home, like how to assemble kitchen appliances”. (Participant O)
Initiation	“What really bothers me is memory and concentration. It’s like things are not getting done the way they used to […] You don’t get around to the actual execution of things and don’t start doing them until it’s a crisis”. (Participant V)
Speed	“The sluggish thinking has been present pretty much all the time. If I need to think or say something more advanced, or really focus on something, I feel that I can’t manage to think the sentence to the end. It is too tough”. (Participant Y)
Mental fatigue	“It [the mental fatigue] is still present in that I can’t sort things out. I don't know how to sort or start doing things”. (Participant V)

Notably, a capacity to manage cognitive challenges during shorter periods by increasing effort was also reported. This ability was experienced to have preceded and contributed to the state of exhaustion, and to make difficulties harder to detect, even for people in the immediate surrounding or by the means of professional assessment.

Participant B2: Now after working full time for a while, I notice that I get much more sensitive towards sensory impressions. Last night, I became dizzy from watching a panoramic scene on tv [...] I perceived the smell of deodorant strongly [...] I find sounds from people on the bus disturbing and use noise-cancelling headphones.

Participant J: I still have really big problems with my memory. Processing and learning new things are kind of impossible. I’m really good at doing repetitive things, however; stuff that’s in my muscle memory. I still manage to do everything I knew before being on sick-leave […] but I can’t learn many new things and it’s really hard dealing with novel situations. I feel that my memory is a total disaster and very uneven. […]. I used to be extremely good at concentrating. I had no problems focusing and was able to sit for hours listening to a teacher, or reading or watching a film. I never had to think about focusing and I was never distracted. It could be the third world war around me but I could still hear what the teacher was saying. It’s a huge difference compared to now. I perish at conferences or lectures where you have to sit and listen.

Participant A: At its worst, I didn’t really notice cognitive difficulties that much since I was pushing myself to the degree that it really consumed me, but I could still remember.

Participant D: If something is urgent, I really need to concentrate. I get an adrenaline rush and feel that ‘I really have to grasp this now’. I do solve it, but it requires a lot of energy and focus.

Participant B2: They said I almost overachieved during the assessment of my work ability, but had I done it later during the day, at say four pm, it would have been a very differently story. I have the ability to concentrate but not for a long time. I do function, for a while.

When asked directly, several of the participants affirmed at least some remaining symptoms of mental fatigue, although a few expressed difficulties defining the concept or asked the interviewer to explain it; the specific descriptions of the experience of mental fatigue differed markedly between persons. Some associated it with other cognitively related problems, such as poor concentration or sensitivity towards stimuli, whereas other persons described or associated it differently, including notions of an uncompromising need to rest or feelings of emptiness.

Participant Y: Can you define it [mental fatigue]? I consider myself mentally fatigued, but how should I think of it really?

Participant U: Mentally, I become sluggish. I can physically feel having gravel in my eyes and getting wrinkled around them. It’s like having this TV white noise, jelly inside the head. It’s just nothing. And if I lay down, I get so tired that I fall asleep.

Several persons reported remaining difficulties in tasks related to linguistic processing. Aside from problems reading, the participants noted that they had trouble finding words during conversation, possibly reflecting either worsened inhibition ability and/ or a general lowering of processing speed [61]. As for the latter, a few participants mentioned problems with thinking slowly, expressed with a Swedish idiom (“Trögtänkt”), literally meaning slow, viscous or sluggish thought. Notably, however, this phrase may also be used in a sense of being unintelligent and a few persons also explicitly reported difficulty with problem solving or higher-order general executive functions.

Participant G2: In some situations when I’m about to speak, I feel that my head is empty. It’s not even messy, just empty. I can’t find words, no alternatives.

Participant Y: My brain wasn’t following along, and I couldn’t concentrate and I was so slow thinking [“trögtänkt”].

### Theme 2. The bigger picture: Cognitive recovery in context

#### Inner and outer barriers for cognitive functioning

Cognitive problems were seen in the context of the participants’ overall well-being and life situation. That is, factors considered important with respect to ED or health in general were reported to have affected cognition as well. For instance, several participants noted how poor sleep had contributed to cognitive problems first appearing and that insufficient sleep or rest was a hindering factor still today. Most commonly, however, cognitive functioning was associated with stress and exhaustion. Cognitive difficulties were reported to having first appeared as a result of a straining lifestyle and many experienced that stressful situations or periods still impacted functioning now, years later. Cognitive symptoms were thus often seen as an early indicator of falling back into bad habits or that life had become more busy or burdensome. The nature of the stressors affecting cognition differed between the participants, and included worries or responsibilities either at home, such as for instance financial difficulties or care over family members with health issues. A few participants also experienced health problems themselves, other than ED, which may had added to cognitive symptoms, including psychological problems such as anxiety or depression, seen either as a part of ED or as factors interacting with it. Some participants particularly emphasized the importance of stressors in the work context as it was considered a more cognitively challenging setting where failures had more obvious consequences.

Participant L: 24 hours weren’t enough so you start to underprioritize sleeping and work late at night. Eventually you’re in a position where the body shuts down so much that neither memory nor a healthy way of thinking works.

Participant M: I’m sensitive in the sense that my ability to concentrate is very closely related to my energy levels. I have to sleep properly, otherwise I lose it.

Participant I: I lose concentration much easier. If I’m in a more stressful period, then I feel I have to stop and sort things out, otherwise it just flutters away.

Participant C2: As soon as I misplace a pair of gloves, I know that I have too much on my plate.

Participant F2: Some days I feel I get less social and have problems focussing and getting things done. Then I need to back up a little bit. For me, it wasn’t just exhaustion but also a part that was depression. I still find dealing with recurring depressions more difficult, although they’re not as bad as they used to be.

Participant G: Cognitive difficulties primarily affect work. At home, no one is afflicted if I don’t do the dishes that day.

#### Cognitive functioning is facilitated by general recovery

Given that cognitive functioning was affected by stress and health, difficulty to differentiate between cognitive improvement and general recovery was noted. For instance, several participants reported that changes made to the overall life situation had lessened stress and cognitive problems alike. This meant that cognitive difficulties had been reduced by factors such as a stronger private economy, less problems in the family, or by changing occupation or employer. Some participants specifically emphasized the benefits of working under a new boss or management, often described together with an experience of being listened to and taken seriously. Others mentioned benefits of new insight and skills learnt during rehabilitation or other therapies. This also included generally health promoting behaviours, such as eating healthy, physical exercise, engaging in pleasurable activities and/ or being outdoors.

Participant R: I think it’s hard to say what I’ve done in order to help just the cognitive things […] When I take micropauses and do my breathing exercises, I don’t think that it’s for my cognition but rather for not feeling so exhausted, and to feel fresher in the brain.

Participant F2: The big thing was going from an employer that didn’t like staff to one that did.

Participant Y: At the Stress-clinic, I was helped in finding all the tools for taking care of myself in a better way: how to do and think, and when to paus and breathe.

Participant A2: What always helps [memory and concentration] is being outdoors. The nature gives me energy and during the summer I tend to my garden […] It’s a lot of work but it’s fun to work the soil. I feel good doing it.

### Theme 3. Overcoming challenges: Strategies for coping with cognitive symptoms

#### Individual strategies

Cognitive recovery was facilitated through the use of various strategies meant to strengthen one’s ability or to compensate for difficulties. For instance, the participants commonly highlighted the usage of external memory support such as calendars and digital reminders, or to-do lists and other forms of note taking. This was often considered strictly necessary as the ability to keep future plans active in memory had become harder, and was related to a general strategy to closely follow pre-decided routines or plans. Similarly, participants also reported of benefits from using mnemonic techniques in order to enhance or regain memory functioning such as by making rhymes or different forms of visualization practices, including the Loci-method. A few persons also noted that hearing information rather than only seeing it was helpful, such as when using sound books instead of reading, or the act of reading out loud instead of in silence. With respect to upholding cognitive functioning, particularly concentration, some persons highlighted the utility of focussing on one thing at the time or giving oneself more time to manage cognitive undertakings.

Participant I: Clearly, my memory is worse now, I have to write everything down. I’ve learnt to use this phone, the calendar in it is excellent. It beeps and reminds you of something you need to do the next day; stuff like that is almost impossible to keep in mind […] My memory really is worse. I live in my calendar and with post-its.

Participant J2: I write plans for myself and for the workplace. I really write them as if one had lost all memory faculties; detailed routines that I'll be able to follow even if I haven't worked on that particular task for a long time. So I guess I have created support myself […] I think in pictures and make rhymes if need to remember say 10 things, like we did in the RECO study, and I’m using the technique where you imagine rooms in your house.

Participant K: I notice that it works better for me now to read out loud, even at work. […] I understand it better if I both read and hear […] Working with something for two hours requires markedly less energy than if the telephone rings and I need to do something in between, for one hour.

Participant K2: If I'm reading up on a subject, I have to give it more time and read it multiple times.

Some persons highlighted the use of strategies intended to train or challenge cognitive abilities which entailed, for instance, returning to former, more active habits or engaging in cognitively demanding tasks in everyday life. Training was described in general terms, but a few participants also specifically noted that the computerized cognitive training programme conducted during the RECO-study had been beneficial.

Participant U: I’m trying to train my brain mentally: crossword puzzles, reading and learning new things in order to keep the brain active. And training one’s balance is really important for motor functioning and the body. If I hadn’t tried to train that, I’m not sure what it would be like now.

Participant J: My memory is obviously better, compared to the absolute beginning, and above all, I’ve learnt strategies. I think it’s a little better since I’ve trained my memory, so to speak.

Participant F: I remember it [memory and concentration] became better just from sitting down doing it [the cognitive training].

#### Optimizing outer conditions

Another set of strategies aimed to improve the conditions under which cognitive tasks were performed, often by planning or organizing life in a way that sufficient resources, or energy, could be directed towards cognitive undertakings. This involved, for example, sleeping well or not doing too much after the workday was over, or to schedule physical exercise or rest, including relaxation exercises or brief pauses which were found useful by many participants. Relatedly, several persons reported of measures taken to improve the work situation with respect to cognition. This included doing less or fewer demanding tasks, or working less or fewer hours. It also involved arrangements enabling more flexibility or control over one’s schedule and environment, and/or a reduction of stressful disruptions and social interactions. On this note, some participants reported of cognitive benefits from not having to share an office or from the option of working remotely. A few participants also noted that the use of noise cancelling headphones had been beneficial for shutting out unwanted disturbances.

Participant D2: [A strategy that helped cognitive functions] was being able to go aside for a moment and just breath. I got a watch that I used to remind me to do that every half an hour, but now it’s set to once every hour.

Participant F: I’ve made a change so I don’t have as long workdays.

Participant E: I’m so much more effective working from home since I don’t have to be around people. It allows me to concentrate. I don’t have the opportunity that often, but when I do, it works really well.

Participant O: One thing that really helped was buying these noise reducing headphones. During one period, I used them a lot. When I got rid of all noise, it was like the brain could relearn and after [a while], I stopped hearing the background sounds.

### Theme 4. The approach towards cognition matters

#### Worry and distress relating to cognitive functioning

It was commonly described how cognitive problems had previously been highly distressing, and some participants still found remaining cognitive symptoms worrying. Several persons had experienced that the brain had been irreversibly altered*,* or compared their functioning to other conditions, such as ADHD or dementia, which may have contributed to a stressed state and made cognitive functioning even more difficult. In contrast, some participants described how they had stopped believing cognitive difficulties to reflect more severe disorders or neurological problems, or that cognitive failures were just not as worrying anymore.

Participant C: Forgetting something that I just said or should be doing, or that I suddenly lose words for things or people that I’ve known for a really long time […], that’s a bit scary.

Participant G: Somehow, It feels like your brain has changed and that you won't be like before.

Participant V: If I had three weeks off, I would just go someplace where there’s peace and quiet; where nothing moves or is heard. I would just lay down to rest and maybe read a book. Somehow, that’s what I’m longing for: the things that don’t exist at home. […] When I talk to someone and there is a small digression in the conversation, I immediately forget the main track. […] To change between thoughts and still manage to remember, that’s hard. It’s really annoying and scary. A while back, I called the health centre and asked to be assessed for Alzheimer’s.

Participant A2: I used to be able to swiftly read a recipe through and then just follow it. Now, I have to go back constantly, and then I start thinking about something else. ‘Did it say 150 grams, and what happens next’. It’s so annoying and irritating, and that makes it take even longer. It relates to becoming even more stressed. I feel like an addle-headed old woman. […] When I feel better and there is less worry, it [the concentration] is better. I guess it's because of the thoughts running. The situation where I feel much better is when on vacation where I can’t really affect whatever worrying situation that might be going on back home.

Participant K2: Back then, I read an article about how depression and exhaustion could leave traces in the brain, so I thought that it would be like that constantly. It felt good to see it actually getting better.

Participant A: When forgetting things now, I no longer think that I'm crazy or me getting stressed about something is making me psychiatrically ill. Now, I rather think: ‘Oh well, maybe I'll remember it another day'.

#### Acceptance, self-compassion, and -prioritization: a shift in perspective facilitates cognitive functioning

During the recovery process, several participants had developed a generally kinder, more acceptant and self-compassionate view of themselves and their problems, as opposed to a previously held more self-critical stance. This shift involved a new approach towards cognition, described by some participants to have helped reduce distress and worry over cognitive failure, and in a broader sense to have facilitated cognitive recovery*.* Notably, acceptance sometimes meant acknowledging a loss of cognitive abilities, and a more self-compassionate approach was related to becoming generally better at prioritizing the own needs, and to set limits and saying no. Still, several participants reported how cognition had benefitted from lowering demands and avoiding too much responsibility or engagement in their work or other tasks.

Participant E: One strategy is to try not becoming too stressed over the fact that I can’t do something. When it happens, I can choose the reaction. And then I try to be gentle with myself […] I’ve noticed that it [the cognitive problems] will pass quicker if I just accept it for what it is.

Participant M: A part of getting well from exhaustion is managing to keep life going. It is to accept that one is different. I’m not as sharp as I used to be: I don’t think as swiftly and I perform worse intellectually.

Participant Q: When I lower the demands, feeling that I’ve done decently at work, or that is ok to have an untidy home, I notice that I have more energy and that my concentration and other cognitive symptoms get better.

Participant B: One thing that’s been helpful is being aware: I’ve understood what recovery is and how I function and the behaviour that makes me put in too much of an effort; that it’s ok to say no.

Participant B2: I'm much kinder to myself. Even if I still try to do a good job, I don’t try as hard to be good in the boss’s and other people’s eyes. I don't have to show it and I actually allow myself to be less ambitious [...] I do notice [that it affects the cognitive abilities]. I think I do it not to crash completely now when I work full-time. Being a little more laid back saves me.

A newfound self-compassionate approach was also related to a conceptual change regarding cognitive difficulties. Specifically, a few participants noted how cognitive problems were increasingly viewed as less of a matter of personal capability and more as a product of a suboptimal environment. Hence, a shift in approach also entailed seeing that the key to cognitive recovery may lie in contextual changes rather than individual improvement. That is, cognitive recovery was no longer merely seen as the boosting of one’s personal performance but rather about knowing the own limitations, saying no, and to actively find an environment that fits one’s needs, instead of the other way around. This notion was emphasized by one participant (J) who recalled something she had said during a meeting with her employer: “To be able to keep working here, I need to have my own room, and control and independence. I can’t constantly adjust myself to other people, they need to adapt to me”.

Participant T: I was pretty hard on myself before all this, feeling that I was weak as a human being, and that everyone around me manages these things. But when I got to meet others in the same situation as me, I understood that I’m not alone feeling like this. Seeing that good people felt the same thing as me enabled me to let it go. Realizing that I’m not worthless but that it’s something that happens to other good people as well […] helped me stop feeling that it's something wrong with me and that it was something that I’ve caused myself. Instead, I got another perspective on my work situation and could see that it wasn’t a good environment.

Participant N: [What have been helpful are] strategies and my general change in approach. I would like to change it a bit more. It’s having Luther on my shoulder [Swedish idiom denoting a strict work ethic]. […] The difference is that I used to say yes to anything. It could be going to a movie or a restaurant when I really wanted to just stay in. Now, if I feel that it won’t give me anything, I’ll just skip it.

Participant P: I keep repeating the same solution but I think it’s really important to be forgiving and to allow yourself not to perform as well. I’ve identified so strongly with being someone who can do everything, managing most things, so when it doesn’t work you become very scared and feel worthless over not managing even the simplest thing. Now, I feel that if I can’t concentrate enough to do the work I've got, then I can do something else. What’s the point, and what kind of match with my life is it, staying at a job that demands something that I can’t handle?

## Discussion

This study explored the experiences of cognitive functioning and recovery in people previously diagnosed with stress-related exhaustion disorder. In sum, the results revealed varied experiences of cognitive functioning 6-10 years after treatment. Overall, even when functioning was improved, remaining symptoms were seen in different domains, reflecting difficulty maintaining concentration and executive control over time. At the time of the interviews, participants experienced that cognitive functioning was still affected by fluctuations in stress, well-being and the general life situation. Hence, means to alleviate stress and promote a generally healthy lifestyle were considered facilitators for cognitive recovery, as were strategies aimed to compensate for, or challenge, cognitive difficulties, or to optimize conditions for cognitive performance. Some participants had adopted a new approach towards cognition marked by self-compassion and acceptance which had facilitated cognitive function. This had also influenced the view of cognitive failure and recovery, which were now seen less in terms of individual aptitude and more as products of external circumstances.

On a more detailed note, the first theme “It’s different now”: Remaining cognitive symptoms*”* showed that at the time of the interviews, the experience of cognitive functioning varied between individuals. Cognitive difficulties, reported to be highly prevalent and distressing when at its worst, were now perceived to be less frequent or disturbing than before, albeit with remaining symptoms. The lingering problems were described in multiple cognitive areas. Nevertheless, executive control was a part of the remaining difficulties across different domains. This included problems with learning or doing new things as opposed to well-known automated tasks; multitasking or managing interruptions or sensory impressions; and reliance on external memory support. With respect to the latter, we have previously seen how this group reported relatively more self-cued than environmentally cued memory failures during an earlier phase of the disorder [46]. The results thus suggests that this pattern may be a long-lasting characteristic of cognitive dysfunction in ED, at least for some people. Moreover, cognitive undertakings were considered achievable but tiring; performance was hence difficult to maintain over longer periods of time. Overall, these reports align with findings from previous research using either cognitive testing or more subjective measures of cognitive functioning. Testing has indicated that cognitive problems in ED may reflect a deficit in executive control, affecting performance in a broad range of domains [26]. It has also been suggested that persons diagnosed with ED may be able to uphold cognitive performance while being distracted, but during a restricted period and at a cost of being more tired or cognitively fatigued, compared to controls [29, 38]. Moreover, follow up-studies in persons with ED or stress-related exhaustion have revealed improvements in cognitive performance across time, but still worse performance compared to healthy controls [37, 48], even decades after onset [32]. As for the subjective experience of cognitive problems, two recent studies are specifically notable. Ellbin, Jonsdottir, and Bååthe [21] revealed that seven to twelve years after the onset of ED, persons still experienced remaining problems in, for instance, maintaining concentration and with multitasking, word-finding as well as with hypersensitivity to external stimuli, largely echoing the results of the current study. Ellbin, Jonsdottir et al. [22] further showed that persons who still fulfilled the criteria for ED reported more problems with learning and memory (including dependency on external memory support, communicating with others, and executive functions, compared to fully recovered ED patients; both groups expressed more difficulties than healthy controls. It is also worth pointing out that previous studies have seen differences in which cognitive domains that are being affected in ED [15, 26], perhaps due to overall heterogeneity in the diagnostic group [43]. Such variability is demonstrated in the current study, where the experiences of cognitive functioning and recovery differ between individuals. However, as indicated by the current results, an alternative interpretation is that difficulty with executive functioning is a domain-general symptom after ED, affecting and interacting with multiple cognitive areas and the experience of fatigue [29]. Possibly, such executive dysfunction is particularly hampering vigilance in a busy or distracting environment, as reported by participants in this study.

With respect to hinders and facilitators, the second theme, *The bigger picture: Cognitive recovery in context*, showed how cognitive problems were seen as intrinsically related to health and the overall life situation, with cognitive difficulties sometimes being thought of as an early indicator of a too-straining life situation. In other words, what had helped or hampered ED recovery, stress in particular, had also affected cognition. We have previously discussed the experience of ED recovery in this sample, including individual facilitators and hinders, in detail elsewhere [2]. Briefly, recovery from ED does not appear as a linear course where one solution fits all, but rather that different kinds of recovery activities and contextual changes are helpful for different persons during distinctive phases of the process, which likely applies to specifically cognitive problems as well. Nonetheless, the reported benefits from active engagement in physical exercise, pleasurable activities, or of reducing stressors in the environment, are broadly in line with previous research on cognitive health factors [51] and should not be overlooked as facilitators for cognitive recovery in ED.

The third theme, *Overcoming challenges: Strategies for coping with cognitive symptoms*, disclosed how cognitive recovery and functioning was also facilitated by the use of compensatory and/ or restorative, individual strategies, including mnemonic techniques, giving tasks more time and - most prominently - the use of external memory support such as electronic reminders, calendars and note taking. This result accords with previous studies showing former ED patients to experience cognitive strategies as being helpful or necessary [21, 22], suggesting that external memory support in particular may be a particularly important feature of cognitive recovery in this group. Moreover, some participants noted benefits from active engagement in cognitively demanding tasks or from cognitive training. We have previously presented promising results of a computerized cognitive training protocol (the same programme mentioned by a few participants in the current study), possibly associated with altered neural, striatal activity [27, 28]. Hence, the current results support that cognitive training may be a viable treatment for at least some persons suffering from ED. The findings are also broadly in line with research on aging populations, showing associations between a cognitively active lifestyle and a more positive trajectory with respect to age-related cognitive decline (e.g., [17]). It is important here to emphasize that while some studies have shown neural alterations in persons with ED, the literature has not provided evidence for any neurological impairment akin to dementia [43], but see also [32]). Nevertheless, it is conceivable that the compensational or reserve-related processes that may help to prevent or slow down decline in aging [8] are involved in the strengthening of cognitive abilities across populations, including ED [28, 46, 49]. We recommend future studies to investigate these processes systematically, possibly in relation to cognitive fatigue [3].

Strategies noted to have facilitated cognitive functioning also involved optimizing the conditions under which cognitive tasks were performed, including various flexible work arrangements such as working from home or adjusting work hours. This aligns with studies showing that such measures, increasingly popular after the Covid-pandemic, may have positive effects on cognition and mental health (e.g., [53], but see also [39]). Overall, means of becoming less distracted during the work day and strategies involving various types of energy management, such as strict adherence to routines regarding sleep, rest, and taking pauses were seen as beneficial with respect to cognitive functioning. This is generally consistent with cognitive load theory [4, 56, 57]. It is also in line with previous findings revealing that persons with ED report higher levels of fatigue from distractions during cognitive testing [29, 38], and show partly different autonomic activity during prolonged test procedures and lesser improvement in performance from repeated testing, compared to healthy controls [29]. In this light, the current results suggest that reducing everyday distractors and not sustaining cognitive activities for too long may, for some persons, be important aspects to consider during cognitive rehabilitation.

The fourth theme, “*The approach towards cognition matters”,* disclosed that some participants still experienced cognitive problems as distressing or compared them to, or worried over, that they reflected other, severe conditions. It is possible that such worry is related to general recovery in ED, as some of the participants who expressed worry or distress over cognitive slips and failures also reported on a still burdensome life situation. It was also noted that worry over cognition had eased, or would ease, in a less stressful setting, such as when being on vacation. In any case, worry and catastrophizing may be seen as adding to cognitive load and likely to hamper cognitive performance for that reason [33]. The finding also resonates with process-based psychological models of stress and cognitive dysfunction, proposing that cognitive functioning is intricately related with multiple factors, including controlling or regulatory behaviour such as rigid worrying or rumination, in turn influenced by a person’s values [11] or metacognitive beliefs or strategies (e.g., [9]). Acceptance and similar concepts are considered means to help facilitate flexibility and to alleviate worrying, and by consequence, exhaustion and cognitive problems [12, 62]. It is therefore noteworthy that some participants reported how, during the rehabilitation process, they had developed an overall kinder, more acceptant and self-compassionate view of themselves. The new approach was experienced to have facilitated cognitive functioning by a reduction in worrying, and a change in perspective. This entailed viewing cognition more as a contextual phenomenon where cognitive failure is considered more a product of an overall bad or stressful situation, rather than merely poor individual performance. Previous research has shown that similar reframing and change of perspective are important experiences ED recovery in general [21], and the current results indicates that this applies also to cognitive symptoms specifically. The findings also suggest that cognitive recovery needs to be seen in context of other symptoms, such as for instance extensive worrying, and that cognitive rehabilitation should address not only overtly cognitive symptoms, but also attitudes and broader beliefs about cognition and oneself.

Finally, this study explored the subjective experiences of people priorly diagnosed with ED. As the study is based on critical realism, it is worth noting that these observations are situated at an “empirical” ontological level, not providing direct insight to “real” level causal mechanisms [14, 41, 64]. Nevertheless, the subjective experiences highlighted here may still indicate “actual” or “real” level processes, and have implications, such as suggested throughout this discussion section. In order to optimize the approximation of cognitive functioning in ED, future research should weigh such experiential information against evidence provided by other forms of empirical cognitive measurement. This includes standardized testing and brain-imaging (implicating specific functions and networks), as well as questionnaires on everyday functioning (better equipped to discern the level of cognitive dysfunction at the group level).

### Limitations and strengths

Some limitations of this study need to be addressed. Firstly, attrition was relatively high between the RECO-project’s first assessment point and the current interviews. Although dropout analysis did not show any group differences between the here included participants and the initial sample with respect to central demographic and clinical variables, it cannot be ruled out that the remaining participants differed in unknown ways, possibly hampering transferability to the larger group of ED patients. On this note, the majority of the sample were women, which holds true for the ED population as a whole [24] and should be considered when interpreting the results of this study. Moreover, the participants have not given feedback on the thematization which may threaten the empirical anchoring of the results. Furthermore, the last seven interviews were conducted during the Covid-pandemic in accordance with restrictions regarding social distancing, with unknown impact on the results. Additionally, despite that the scope of this study was to explore cognitive functioning broadly, the participants were specifically prompted about memory, concentration and mental fatigue. While these areas are included in the diagnostic criteria and of interest for that reason, it is possible that the results would have been somewhat different had the interview guide exemplified cognition with other domains. Although we acknowledge this as a methodological limitation, it should be noted that the participants were asked additional open-ended questions about general ED recovery, enabling expression of important experiences of cognitive functioning in any domain, which was likely reflected in the heterogeneous results. Similarly, it is also worth seeing that this study has categorized the participants’ descriptions of their functioning using terminology from the field of cognitive psychology (e.g., inhibition or working memory). While this approach may provide relatively detailed information on the experienced difficulties, which is a strength, a risk is that the thematization may not capture the verbatim descriptions by the individual participants, normally worded in non-technical, everyday language. We argue that such discrepancy may be inherent in all thematization, but it should nonetheless be considered when reflecting on the current results. Regarding terminology, possible incongruence between psychological theory and everyday language is also conceptually interesting, and should be further investigated by future research.

The study also had some methodological strengths, including a well-defined population and a relatively large number of participants which has helped to provide rich data. Also, overall, the interviews were coded and interpreted in close proximity to the participants own accounts and showed a range in features and responses that are likely inherent to the larger group of persons diagnosed with ED [42], thereby enhancing credibility and transferability of the results. Moreover, in our view, a strength of this study is its transparency and reflexivity regarding the theoretical context and the authors’ pre-understanding. Although the bulk of the coding was done by the first author, a possible limitation in itself, the procedure was frequently discussed with the other authors, most of which have been previously engaged in research on ED and cognition. Two authors (HMG and IA) are also currently active as clinical psychologists at the stress-clinic where the participants were recruited and interviewed. While this may have increased the risk of confirmation bias, we nevertheless believe that the engagement in the field and knowledge about the patient group has helped to enhance credibility. On this note, the contribution of an author from a different research background (MT), has also been valuable in providing perspective.

### Summary and implications

This qualitative study shows a range of experiences regarding cognitive recovery after ED. Importantly, also when cognitive functioning is noted to be better, 6-10 years after treatment, it is still interwoven with the overall context, including work conditions, stress and well-being. With respect to clinical practice, this indicates that cognitive functioning may for some people be improved as a function of general stress-reducing or mood-lifting interventions. However, it moreover suggests that in order to make such treatment effective, potential lingering cognitive difficulties should be considered and clearly addressed in rehabilitation. This poses a clinical challenge as there are marked individual differences in symptomology and in which strategies that are being perceived as helpful. In other words, the results suggests that one size does not fit all, and a need for personalized rehabilitation in accordance with precision health practice (e.g., [34]). That is, some persons may improve functioning through a reduction in cognitive load, using external cognitive support or by not sustaining tasks for too long. Others might benefit more from active engagement in challenging cognitive undertakings, or from therapeutic techniques aiming to facilitate acceptance or alter metacognitive beliefs [12, 63], which should be further evaluated in the context of ED. The choice of intervention may depend on the current life situation and in which stage of the recovery process that a patient finds herself in (see Aronsson et al. [2] for a general discussion on stages of recovery), but also on other individual differences. Hence, we recommend clinical practice to actively assess cognitive functioning rather than to assume a specific cognitive profile or course in ED. Moreover, as noted in other clinical groups, strategies or enhancement of cognitive functioning should not only be learnt or talked about in the clinic, but purposely transferred to a person’s unique everyday life situation [19].

Regarding cognitive assessment, this study has shown how difficulties in relatively complex everyday tasks may be experienced and allocated to a wide range of specific domains. Therefore, both clinical practice and further research should aim to assess cognition broadly, but make sure to include measures of executive functioning or attention, which may be permeating features across domains. Moreover, as the experience of cognitive difficulties may be heterogeneous in the ED population, future studies are recommended to investigate the cognitive sequala of ED, and its neural substrate, with methods or designs allowing differentiation between individual patients or perhaps sub-groups of patients in different phases of recovery (e.g., [40]). Furthermore, we propose that both future studies and clinical practice ought to consider including measurement that extends traditional, neuropsychological testing. These procedures should be able to provide important nuances of individual functioning, such as the impact of longer task duration, distinctive types of stressors and distractions, and external memory-support. Semi-structured interviews may be well equipped to provide additional information on the subjective experience of such manipulation, metacognitive beliefs or values, and the circumstances under which cognitive performance are seen as manageable rather than problematic.

Finally, the results of the current study indicates that cognitive difficulties can be distressing, long-lasting and should be taken seriously. Importantly, this study also highlights that across persons, there are multiple facilitators of cognitive recovery and that many experience that it does get better with time, which is hopeful and should be clearly communicated.

## Supplementary Information

Supplementary Material 1.

## Data Availability

The datasets generated and/or analysed during the study are not publicly accessible due to Swedish law (the Swedish Ethical Review Act: 2003:460) but are available from the authors on reasonable request. For such requests, please contact Anna Stigsdotter Neely or Hanna Malmberg Gavelin.

## Abbreviations

ED: Exhaustion disorder
ICD: International Statistical Classification of Diseases and Related Health Problems
SCCs: Subjective cognitive complaints
RECO: Rehabilitation for Improved Cognition
PRMQ: The Prospective and Retrospective Memory Questionnaire
MMR: Multimodal Stress Rehabilitation
CBT: Cognitive Behavioural Therapy
SMBQ: The Shirom-Melamed Burnout Questionnaire
s-ED: The Exhaustion Disorder scale
HADS: The Hospital Anxiety and Depression Scale
WAI: Work Ability Index
ACT: Acceptance and Commitment Therapy
