# Supplementary material for: The experienced route to cognitive health: Cognitive recovery in persons with prior stress-related Exhaustion disorder
Source: BMC Psychiatry. 2025 Apr 14;25:375. doi: 10.1186/s12888-025-06713-7 (PMC11995536; doi:10.1186/s12888-025-06713-7)
Supplement: Supplementary file 1 — Supplementary Material 1. [file 12888_2025_6713_MOESM1_ESM.docx]

**Additional file 1**

**Appendix 1. Diagnostic criteria for exhaustion disorder (F43.8A) according to the Swedish National Board of Health and Welfare (ICD-10-SE)**

A. Physical and mental symptoms of exhaustion during at least two weeks. The symptoms have developed in response to one or more identifiable stressors, which have been present for at least 6 months.

B. Markedly reduced mental energy, manifested by reduced initiative, lack of endurance, or increase of time needed for recovery after mental efforts.

C. At least four of the following symptoms have been present most of the day, nearly every day, during the same 2-week period:

1. Persistent complaints of impaired memory and concentration

2. Markedly reduced capacity to tolerate demands or to perform under time pressure

3. Emotional instability or irritability

4. Insomnia or hypersomnia

5. Persistent complaints of physical fatigue and lack of endurance

6. Physical symptoms such as muscular pain, chest pain, palpitations, gastrointestinal problems, vertigo, or increased sensitivity to sounds

D. The symptoms cause clinically significant distress or impairment in social, occupational, or other important areas of functioning.

E. The symptoms are not due to the direct physiological effects of a substance (e.g., abuse of a drug or medication) or a general medical condition (e.g., hypothyroidism, diabetes, infectious disease).

F. The stress-related disorder does not meet criteria for major depressive disorder, dysthymic disorder, or generalized anxiety disorder.

**Appendix 2. Flow of participants in RECO-trial**

**Appendix 3. Memory aid**

**
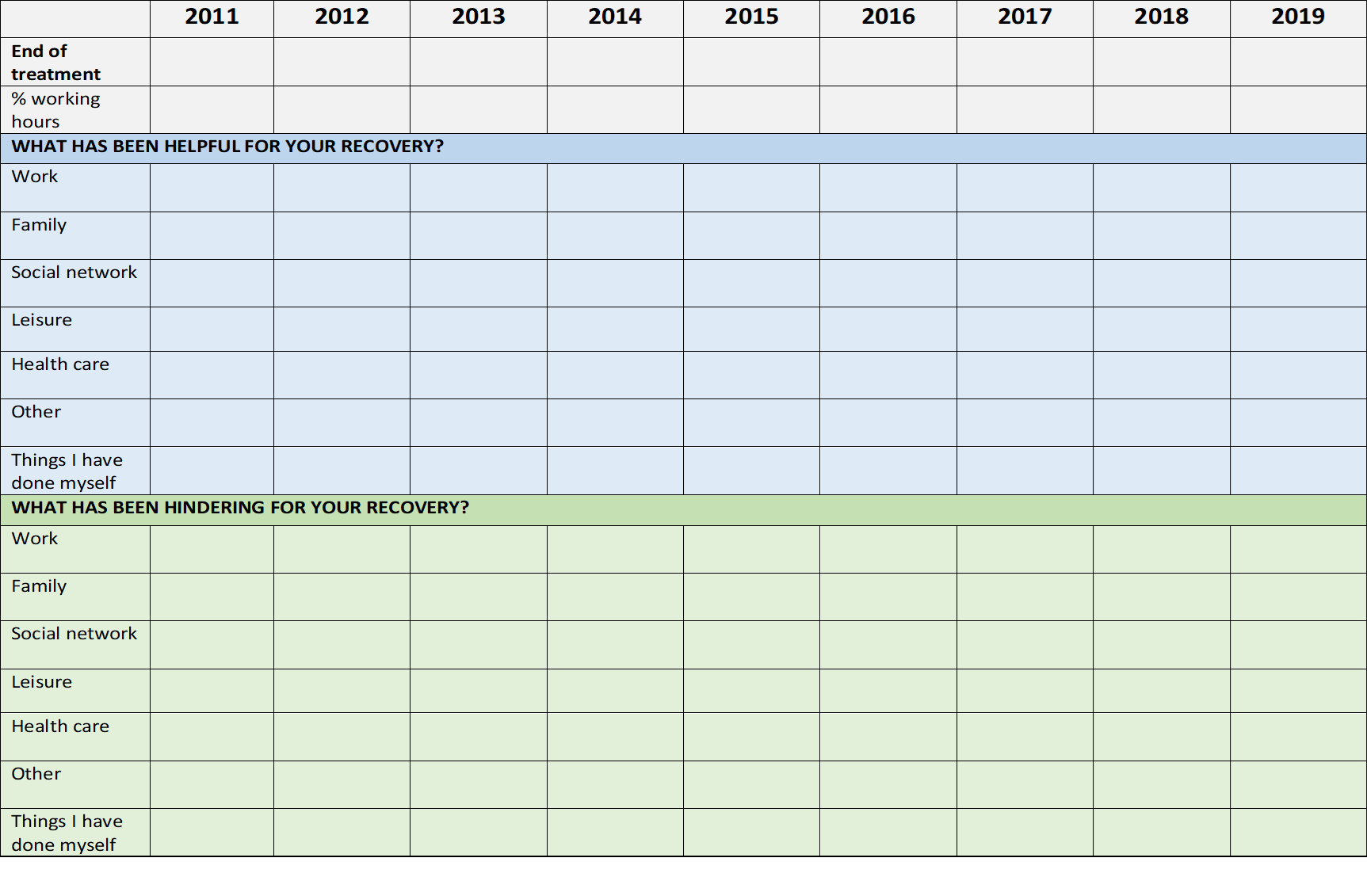
**

**Appendix 4. Interview guide**

**Recovery and recuperation**

- How are you feeling today in relation to your previous exhaustion disorder?
- Based on the answers to the questionnaire: Could you briefly describe the events that you feel have been helpful for recovery from exhaustion disorder since the end of treatment?
  - If noted, briefly describe what has hindered recovery.

**Recovery cognition**

- Based on the questionnaire, follow up: How is your memory/concentration/mental fatigue functioning today?
  - *Examples?*
- If there is improvement: what has been helpful?
  - *Were any adjustments made at the workplace that were helpful?*
  - *Any own strategies that have been helpful?*
- Obstacles/things that made it difficult?
- *Anything missing that could have helped?*

**Recovery behaviors and activities**

- What are you doing today to take care of yourself in relation to your previous exhaustion disorder?
  - What do you experience as restorative?
- What did you need to do to take care of yourself in relation to your exhaustion disorder when you were the most ill?
  - Has the need for recovery changed?
  - Are the same things restorative today as they were then?
  - Difference in the effect of different recovery behaviors?
- What are your thoughts about the importance of the workplace in creating opportunities for recovery at work?
  - Any adjustments in the workplace that have influenced your opportunities for recovery?
  - Anything missing that could have been helpful (from the employer)?
  - Obstacles/things that have made it difficult?

*Note*. The questions of primary interest to this study are underlined.

**Appendix 5. Summary of initial interpretation and conceptualization of cognitive domains**

| **Cognitive function** | **Meaning/ definition** | **Examples** |
| --- | --- | --- |
| 1. Episodic memory | Ability to learn, storage and retrieve information across an extended period of time |  |
| 1.1. Prospective memory | Memory for future intentions”/ Ability to remember to do things in the future | Failing to remember to take medication.  Forgetting planned appointments. |
| 1.2. Retrospective memory | Recollection of past events | Forgetting something previously learnt or told  Mislaying things, e.g. glasses or telephone |
| 1.3. Self-cued memory | Remembrance in situations where memory retrieval is not obviously cued by external reminders or triggers in the environment. | Forgetting appointments if  not prompted by someone else  or by a reminder such as  a calendar or diary. |
| 1.4. Environmentally cued memory | Remembrance in situations where memory retrieval is cued by external reminders or triggers in the environment | Failing to recognize a  place visited before.  Forgetting to buy something  that has been planned to buy, like a birthday card, even when seeing the shop. |
| 2. Concentration/ focus general | Difficulties focussing or concentrating, not further specified | Can’t concentrate |
| 2.1. Vigilance | Difficulties concentrating over time | Focussing over longer time periods does not work  Can’t follow an entire movie  Endurance is not there anymore (with respect to concentrating) |
| 3. Executive functions | A collection of top-down control processes used when  going on automatically or relying on instinct or intuition would be ill-advised, insufficient, or impossible. |  |
| 3.1 Inhibition | Controlling one’s attention, thoughts or memories to override a strong internal predisposition or external lure. |  |
| 3.1.1 Interference control | Ability to focus on a specific task and not be distracted  Includes selective or focused attention; inhibition of thoughts and memories. | Difficulties concentrating when interrupted.  Easily distracted (either internal or external, e.g., by sounds or unwanted thoughts).  Needing to re-read text when distracted. |
| 2.1.2 Response inhibition | Inhibition at the level of behaviour – response inhibition. | Acting impulsively or prematurely  Difficulty resisting temptation |
| 3.2 Working memory | Holding information in mind while mentally working with it (e.g., relating one thing to another, using information to solve a problem). Including mental math, reordering  items, or relating one idea or fact to another. | Trouble with tasks that have more than one step  Forgetting instructions easily  Difficulty reading (e.g. missing out with respect to the context in the text, rather than losing concentration or being distracted)  Trouble translating instructions to action (e.g., following a recipe) |
| 3.3 Flexibility | Changing perspectives or approaches to a problem, flexibly adapting to new demands, rules or priorities, switching between tasks  Including being able to “think outside the box,” see something from many different perspectives, quickly switch between tasks, or flexibly switch course when needed | Trouble changing from one activity or task to another  Difficulties dealing with unexpected changes  Difficult to re-focus when interrupted (e.g, someone comes into the office)  Problems doing two things at the same time (e.g., making dinner and helping your children with their homework)  Trouble finding/accepting alternative solutions to a problem |
| 3.4 Higher level executive functions | Reasoning  Problem-solving  Planning  Organization | Difficulty drawing conclusions  Difficulty finding solutions to problems  Difficulty organizing behaviour in in order to reach a goal  Difficulty achieving (relatively long-term) goals through intermediate steps.  Trouble prioritizing activities  Trouble organizing work  Overwhelmed by large tasks  Starting tasks (e.g., cooking, projects) without having the right materials  Trouble planning and organizing everyday activities |
| 3.5 Initiation | Beginning a task or activity, generating ideas, responses or problem-solving strategies ( | Trouble getting started with activities  Need to be reminded to begin a task |
| 4. Mental Speed | Time it takes to process information, perform mental operations and act on that information | Thoughts are slow  Need relatively long time in order to complete simple mental tasks. |

**Appendix 6. A priori coding template**

1. Lived experiences of current cognitive functioning
   1. Episodic memory (ability to learn, storage and retrieve information across an extended period of time)
      - 1. Prospective memory
        2. Retrospective memory
        3. Short-term memory
        4. Long-term memory
        5. Self-cued memory
        6. Environmentally cued memory
   2. Executive functions (a collection of top-down control processes used when going on automatic or relying on instinct or intuition would be ill-advised, insufficient or impossible)
      1. Working memory
      2. Inhibitory control
      3. Cognitive flexibility
      4. Higher level executive functions
   3. Attention and concentration (ability to focus on a specific task and not be distracted)
   4. Mental speed (Time it takes to process information, perform mental operations and act on that information)
2. Lived experience of change in cognitive functioning
   1. Episodic memory
      - 1. Prospective memory
        2. Retrospective memory
        3. Short-term memory
        4. Long-term memory
        5. Self-cued memory
        6. Environmentally cued memory
   2. Executive functions
   3. Attention and concentration
   4. Mental speed
3. Barriers and facilitators for cognitive recovery
   1. Barriers
   2. Facilitators

**Appendix 7. The final coding template**

1. It's different now_remaining cognitive symptoms
   1. Change in cognitive functioning
      1. Cognitive functioning has changed
         1. CF is better than it was before
            1. CF is better but not as well as before the onset of ED

Concentration better but not as well as before ED

Memory better but not as well as before ED

MF better but not as well as before ED

Working memory better but not as well as before ED

- - - - 1. CF is generally better but has recently become worse again

Concentration better but worse again

Memory better but recently worse again

- - - - 1. Recovered overall but can’t accelerate (“gasa”) as much as before
        2. Concentration better
        3. Memory better

Memory better but is difficult to trust

- - - 1. CF is worse
         1. CF is worse but at a normal level compared to other people
         2. Concentration worse but at a normal level
         3. Memory worse but at normal level
      2. The trajectory differs between cognitive domains
         1. Concentration better but memory not
         2. Memory better but concentration not
         3. Memory and_or concentration better but MF is not
      3. Better now at dealing with cognitive tasks, but haven’t got better cognitive ability
    1. No or low change
       1. No or very few cognitive problems
       2. Poor CF before getting ill_ED
          1. Concentration poor before ED
          2. Memory poor before getting ill_ ED
       3. CF stable
          1. Concentration problems relatively persistent or stabe
          2. Memory problems relatively persistent or stable
    2. CF fluctuates
       1. CF varies with how you feel
       2. CF varies with level of Mental Fatigue
       3. CF varies with level of stress
          1. Concentration dependent on stress
          2. Memory function dependent on stress
       4. CF varies with sleep
       5. CF varies with use of strategies
  1. Type of cognitive problems
     1. Before ED
        1. Domains and situations
           1. Memory

Learning new things

No Problems or good functioning

Problems

General memory, Problems

General memory_No Problems

- - - - 1. General concentration_Focus_Attention

1.2.1.1.2.3 General concentration_Problems

- - - - 1. Executive functions

Flexibility

No Problems

Could multitask before ED

- - - 1. Specific situations or tasks in which cognitive difficulties are noted
         1. Verbal ability_language

Finding words

Finding_remembering names

Reading

Specifically difficult reading fiction

Specifically difficult reading non-fiction

No difficulty reading

Writing

No problems

Second language

- - - 1. Following TV or movies
      2. Doing things with computer
      3. 1.2.1.1.7.4 Cooking
    1. When at its worst
       1. Domains and situations
          1. Memory

Episodic Memory

Prospective Memory

Problems

Retrospective Memory

Problems

Self-cued Memory

No Problems

Short-term memory

Episodic_Problems

Learning new things

Problems

General memory_No problems

General memory_ Problems

- - - - 1. General_Concentration_Focus_Attention

Vigilance

No Problems

Problems

General Concentration_No Problems

General concentration_Problems

- - - - 1. Executive functions

Inhibition

Interference control

Problems

Flexibility

Problems

Multitasking is hindering

Working Memory

Problems

Higher Level Executive Functions

Problems

Initiation

Problems

- - - - 1. Speed

Trögtänkt

Problems

Motor speed

Problems

- - - - 1. Mental fatigue (MF)

MF is defined or associated with different phenomena

Difficulty defining MF

Needs help defining MF

Beliefs or associations to MF

Stimuli-related beliefs or association

MF associated with being sensitive towards too much stimuli

Beliefs or associations related to cognitive phenomena

MF associated with poor concentration

MF associated with spacing out

MF_associated with not listening

Emotion-related beliefs or association

MF associated with lack of joy

MF associated with sorrow and deathwish

MF leads to panic

Brain-related beliefs or association

MF associated with brainfog

MF entails that the brain stop working

MF is associated with the brain being mashed

Stimuli-related beliefs or association

MF is stress induced

Beliefs or associations related to being tired or needing to rest

MF_associated with just needing to rest

Beliefs or association related to other people or upholding personal space_limits

MF comes from meeting people

Beliefs or association related to the rest of the body

MF associated with headache

Problems with mental fatigue

- - - 1. Specific situations or tasks in which cognitive difficulties are noted
         1. Verbal ability_language

Finding words

Finding_remembering names

Reading

Difficulty reading

Specifically difficult reading fiction

Specifically difficult reading non-fiction

No difficulty reading

Writing

No Problem

Second language

- - - - 1. Following TV or movies
        2. Doing things with computer
        3. Cooking
        4. Knowing_Finding directions
        5. Driving
    1. Now_remaining symptoms
       1. Domains and situations
          1. Memory

Episodic Memory

Prospective Memory

Problems

Retrospective Memory

Problems

Self-cued Memory

Problems

Short-term memory

Problems

Long term memory

Problems

Episodic_Poor functioning

Learning new things

Problems

General memory_No problems

General memory_Problems

- - - - 1. General_Concentration_Focus_Attention

Vigilance

Problems

General concentration_Problems

Maintaining concentration

Problems

Concentration_No Problems

Concentration_Problems

- - - - 1. Executive functions

Inhibition

Interference control

Problems

Flexibility

No Problems

Problems

Working Memory

No Problems

Problems

Higher Level Executive Functions

Problems

Initiation

Problems

Non-automated cognitive processing

Automatic_well learnt tasks functions better than executive demanding_novel tasks

Memory functioning no longer automatic_cf requires deliberate attention

- - - - 1. Speed

Trögtänkt

Problems

- - - - 1. Mental fatigue (MF)

MF is defined or associated with different phenomena

Defining MF

Difficulty defining MF

Needs help defining MF

Beliefs or associations to MF

Associations relating to void or emptiness

Associations related to stimuli

MF associated with being sensitive to stimuli

MF associated with TV-noise

Associations related to cognition

MF associated with difficulties sorting out or starting activities

MF associated with poor concentration

MF associated with spacing out

MF associated with thinking many thoughts

MF entails not being sharp

MF entails or appears as difficultues with Vigilance

MF is a response to cognitive performance_demands

Associations related to emotions

MF associated with lack of joy

Associations relating to the brain

MF entails that the brain is not working properly

MF entails that the brains stop working

Associations related to energy, being tired or needing to rest

MF is associated to being (non-bodily) tired

MF associated with just needing to rest

Beliefs or association related to other people or upholding personal space_limits

MF associated with difficulties upholding personal space_integrity

MF comes from meeting people

MF associated with being in a small bubble_shutting the world out

No or markedly less problems with MF

Problems

- - - - 1. Arithmetics or processing numbers

Problems

- - - - 1. Specific situations or tasks in which cognitive difficulties are noted

Conversation_ using language

Finding words

No problems

Problems

- - - - 1. Reading

Problems

No problems

Specifically difficult reading fiction

Specifically difficult reading non-fiction

- - - - 1. Remembering names

Problems

- - - 1. Following TV or movies
      2. Doing things with computers
      3. Cooking
      4. Knowing_Finding directions
      5. Driving
      6. Cognitive problems appear or associated with social situations_interactions
         1. Helpful not meeting people
      7. Sensitivity to sensory impressions
      8. Cognitive problems hard to see
    1. Memory problems hard to see
    2. Cognitive tasks costs much energy_are exhausting

1. The bigger picture_Cognitive recovery in context
   1. Inner and outer barriers for cognitive functioning
      1. CF is worsened or varies in relation to stress, health and how you feel
         1. Cognitive functioning worsened by stress
            1. Cognitive symptoms appear in stressful period or situation

Stressful social situations

CF is hampered by stressful life events

CF is hampered by worry over unemployment_ work ability

Stress_worry over ill family member hinders CF

Concentration worsened by stress

Concentration difficulties appear in stressful periods

Concentration difficulties come from stress

Concentration difficulties come from unexpected stressful events

Concentration difficulties in stressful situations with many people

Memory difficulties appear in stressful periods

- - - - 1. Cognitive problems are an indicator of stress or of having too much to do

Better cognition is a sign of less exhaustion

Cognitive symptoms a sign of too much strain

Forgetting things is a sign of having too much around oneself

Memory difficulties is a sign of stress

Mental fatigue is a sign of doing too much

Mental fatigue is an indicator of stress

Metal fatigue or poor concentration is a sign of not having recovered enough

Poor concentration a sign of changes of changes at work

Poor concentration a sign of more strain or stress

Poor concentration is a sign of exhaustion

Stress is a sign of memory difficulties

Losing words is a warning sign

- - - 1. CF is hindered by poor sleep or feeling tired
         1. Cf functioning worsened by not sleeping well

CF is worsened by stress_worry over not sleeping enough

Need to sleep to function cognitively

- - - - 1. Feeling tired or exhausted is hindering

Memory worse when tired

Concentration worse when tired

- - - 1. CF is affected by other psychiatric or medical problem
         1. Accident_Physical trauma is affecting CF

Worse concentration after accident

- - - - 1. Dyslexia is affecting CF
        2. OCD is affecting CF
        3. CF different in ED than after stroke
        4. Depression or depression-like symptoms affects CF
      1. Cognitive tasks are associated with headache
      2. CF explicitly related to general well-being
    1. CF is hindered by work or home environment
       1. Home Environment
          1. Family or life events is hindering

Care for children with health issues is hindering

- - - - 1. Everyday chaos hinders learning
      1. Work environment
         1. Not much or insufficient changes_adaptation at work
         2. Cognitive functioning to large extent_mostly or primarily a work place problem
         3. Concentration was hindered by noise distractors
         4. Hindering_Poor support or understanding from employer
         5. Hindering_High or too high work load
         6. Hindering_Workplace not allowing recovery or healing
         7. Difficulty focussing in work environment

Distracting or complex work environment

- 1. Cognitive functioning is facilitated by general recovery from ED
     1. Getting professional help is helpful
        1. Care at stressrehab was helpful
        2. Seeing other psychologist, psychotherapy was helpful
        3. Wishes_Individual therapy
        4. More follow up from Stressrehab after return to work
        5. Practical help structurering work
     2. CF is facilitated by health promoting behaviour
        1. Avoiding alcohol is helpful
        2. Being outdoors is helpful
        3. Eating healthy is helpful
        4. Leisure_Engaging in other pleasurable activities are helpful
           1. Photographing
           2. Being distracted_relaxed from TV is helpful
           3. Gardening is helpful
           4. Handicraft is helpful
           5. Dance or music helpful
        5. Physical activity is helpful
           1. Pulse-raising exercise is helpful
           2. Walking_Strolling is helpful
        6. Less computer_screen time is helpful
           1. Not using cell phone_social media as much is helpful
     3. Better home environment
        1. Better financial situation is helpful
        2. Children in a better situation
     4. Better work environment
        1. Changing work or support from employer or boss
           1. Having a good boss_employer
           2. Quitting or changing work_employer
        2. Pleasurable_interesting work tasks
     5. Social or occupational support
        1. Informing others about stress
        2. Meeting others with similar problems is helpful
        3. Other people understanding is helpful
        4. Sharing and communicating with other people is helpful
        5. Meeting colleagues
     6. Giving the recovery process time is helpful
     7. Getting more insight and knowledge
        1. Self-knowledge_Insight on own reactions and behaviour
        2. Knowledge about ED
        3. Knowledge from Stressrehab
        4. Self- help books
        5. Wishes more knowledge_information about cognition

1. Overcoming challenges_Strategies for coping with cognitive symptoms
   1. Individual Strategies
      1. Compensatory strategies
         1. External memory support
            1. Calendar
            2. Electronic reminders
            3. Making lists or taking notes
            4. Asking other people for help remembering
            5. Belief that usage of external memory aids is negative or not proper improvement
      2. Hearing
         1. Soundbook
         2. Reading out loud
      3. Mnemonics
         1. Rhymes
         2. Thinking in pictures_Visualising
      4. Compensating by trying harder or increasing effort
         1. Able to maintain cognitive functioning as long as not relaxing
         2. Shutting emotions off makes you sharper and to focus harder
         3. Sometimes able to maintain focus by putting in more effort
      5. Giving tasks more time
         1. Sleeping on it
         2. More time to think
         3. Doing tasks early_not waiting until the last minute
      6. Doing or focusing on one thing at a time
      7. Managing task by planning or following pre-decided plan
         1. Helpful if tasks are predictable
      8. Challenging or strengthening strategies
         1. Challenging or training oneself cognitively
         2. General training
         3. Cognitive training programme
         4. Engaging in cognitively challenging tasks
            1. Crosswords
            2. Reading
         5. Behavioral activation_returning to old habits
            1. Going back to work
   2. Optimizing outer conditions
      1. Avoiding or reducing the amount of cognitively difficult tasks
         1. Fewer work tasks
         2. Doing less or fewer administrative tasks_writing
         3. Reducing or avoiding interactions or tasks involvning other people at work
      2. Less work or fewer work hours
         1. Shorter days
         2. Working fewer hours
            1. Wishes to work fewer hours
         3. Wishes more or longer breaks during the work day
         4. Wishes more resources_staff at work
      3. Flexibility and control over work situation
         1. Variability in work tasks
         2. Control over schedule_work situation
            1. Flexible work hours
            2. Wishes more control over work situation
         3. Doing pleasurable, fun things at work
         4. Working from home is helpful
      4. Less distracting environment
         1. Not doing cognitive tasks in home environment helpful
         2. Less distracting work environment
            1. Not sharing office
            2. Wishes Peace and quiet
            3. Not having a beeper
         3. Sound reducing headphones are helpful
      5. A structured life and long-term plans are helpful
         1. Following routines for feeling better_lifestyle
            1. Sleep_rest routine
            2. Eating routine
            3. Training routine
         2. Making long-time plans is helpful
            1. Planning the year in advance
      6. Energy management
         1. Taking breaks are helpful

Allocating time for recovery_breaks is helpful

Electronic reminder to take pauses is helpful

A moment to breath is helpful

Micropauses are helpful

- - 1. Resting is helpful
    2. Finding balance with respect to energy is helpful
       1. Slowing down is helpful
    3. Sleeping or sleep routines are helpful
    4. Mindfulness or meditation practises are helpful
    5. Relaxation practices are helpful

1. Acceptance and self-compassion_A shift in perspective facilitates cognitive functioning
   1. Worry and distress relating to cognitive functioning
      1. Worrying or catastrophizing
         1. Comparing with other medical condition
            1. ADHD
            2. Neurodegenerative_dementia

Afraid of dementia

Comparison dementia

- - - - 1. Aphasia
      1. Notion that the brain is not working or has changed
      2. Cognitive problems associated with becoming crazy
      3. Finds cognitive ability difficult to trust
      4. Belief that cognitive problems are not normal
      5. Worry makes cognitive functioning worse
    1. Less worried or distressed about cognitive symptoms now
       1. Not worrying or catastrophizing as much now
          1. Normalizing problems
          2. Catastrophizing to lesser degree
       2. Worrying less overall is helpful
  1. Acceptance and self-compassion A shift in perspective facilitates cngnitive functioning
     1. Acceptance
        1. Acceptance helps CF
        2. Accepting that cognitive ability is not the same anymore
     2. Self_compassion or being kind to oneself
        1. Being kind to oneself
        2. Self_compassion
        3. Prioritizing own needs
           1. Accepting not having to do everything oneself
     3. Setting limits, lowering demands or saying no is helpful
        1. Saying no is helpful
        2. Setting limits is helpful
           1. Reducing or avoiding responsibility
        3. Less demands_engagement or responsibility
           1. Avoiding engaging too much at work
           2. Less responsibility

Wishes less responsibility

Wishes_Less responsibility at work

Wishes_Less responsibility in private life

- - 1. A new perspective on cognition
